# Elucidating the Effect of Etching Time Key-Parameter toward Optically and Electrically-Active Silicon Nanowires

**DOI:** 10.3390/nano10030404

**Published:** 2020-02-25

**Authors:** Mariem Naffeti, Pablo Aitor Postigo, Radhouane Chtourou, Mohamed Ali Zaïbi

**Affiliations:** 1 Laboratory of Nanomaterials and Systems for Renewable Energies (LaNSER), Research and Technology Center of Energy, Techno-Park Borj-Cedria, Bp 95, 2050 Hammam-Lif, Tunis, Tunisia; radhouane.chtourou@crten.rnrt.tn (R.C.); medali.zaibi@ensit.rnu.tn (M.A.Z.); 2 Instituto de Micro y Nanotecnología, IMN-CNM, CSIC (CEI UAM+CSIC) Isaac Newton, 8, E-28760 Tres Cantos, Madrid, Spain; pabloaitor.postigo@imn.cnm.csic.es; 3 Tunis University—National High School of Engineering of Tunis, 5 Av Taha Hussein 1008 Tunis, Tunisia

**Keywords:** silicon nanowires, metal assisted chemical etching, etching time, optical properties, electrical properties

## Abstract

In this work, vertically aligned silicon nanowires (SiNWs) with relatively high crystallinity have been fabricated through a facile, reliable, and cost-effective metal assisted chemical etching method. After introducing an itemized elucidation of the fabrication process, the effect of varying etching time on morphological, structural, optical, and electrical properties of SiNWs was analysed. The NWs length increased with increasing etching time, whereas the wires filling ratio decreased. The broadband photoluminescence (PL) emission was originated from self-generated silicon nanocrystallites (SiNCs) and their size were derived through an analytical model. FTIR spectroscopy confirms that the PL deterioration for extended time is owing to the restriction of excitation volume and therefore reduction of effective light-emitting crystallites. These SiNWs are very effective in reducing the reflectance to 9–15% in comparison with Si wafer. I–V characteristics revealed that the rectifying behaviour and the diode parameters calculated from conventional thermionic emission and Cheung’s model depend on the geometry of SiNWs. We deduce that judicious control of etching time or otherwise SiNWs’ length is the key to ensure better optical and electrical properties of SiNWs. Our findings demonstrate that shorter SiNWs are much more optically and electrically active which is auspicious for the use in optoelectronic devices and solar cells applications.

## 1. Introduction

In recent years, silicon nanowires (SiNWs) have aroused tremendous attention worldwide thanks to the following outstanding features: (1) Environment-friendly as second most earth-abundant materials; (2) unique dimensional structures (1 D); (3) interesting electrical and optical properties compared to bare silicon; (4) affordable fabrication; and (5) potential applications in several fields [1,2,3,4,5]. The various applications of these nanostructures may include lithium-ion batteries [6], biochemical sensors [7,8], electronics [9], catalysis [10], and solar cells [1,11].

Based upon the bottom-up and top down approaches, numerous methods have been used to fabricate SiNWs such as vapor–liquid–solid, thermal evaporation, molecular beam epitaxy, laser ablation, and lithography [12,13,14,15,16]. However, these techniques have some limitations as they generally require expensive and complex equipment, employ hazardous silicon precursors, and involve high vacuum and high temperature [17]. These features make the synthesis expensive and time-consuming and therefore hindered their applications for commercialized products. In contrast, an effective and promising synthetic method namely metal assisted chemical etching (MACE) has been proposed [2,4,18,19,20]. This technique is simple, rapid, low cost, and suitable for both industrial and laboratory scales. Moreover, MACE allows to obtain high crystalline SiNWs quality, as well as an easy control of the different parameters including orientation, doping type, length, and diameter.

The MACE method basically consists of two procedures, the formation of metal catalysts and the subsequent etching process which can be implemented either in a single step (1-MACE) [10,21] or in two steps (2-MACE) [17,18,19,20]. Moreover, the formation method, etching time, etching temperature, metal deposition time, and lastly the etchants’ concentrations have a crucial influence on the morphology of SiNWs [2,5,17]. Ghosh et al. reported that SiNWs grown by MACE are usually covered with silicon nanocrystals due to the side wall etching and which are the origin of quantum confinement (QC) effects owing to their small dimensions [20].

Recently, several research groups have succeeded in the synthesis of optically-active SiNWs exhibiting a significant PL emission and a very low reflectance [3,4,17]. On the other hand, Qi et al. have demonstrated the fabrication of electrically-active SiNWs through heavily doped SiNWs with rough surface where a high Schottky barrier exists at the interface of SiNWs and the metal [22]. Nevertheless, further investigation is required to explore both optically and electrically active SiNWs. Indeed, a number of studies have investigated the optical and electrical properties of SiNWs [23,24,25]. It has been shown that SiNWs, obtained by 1-MACE in AgNO_3_, HF, and H_2_O_2_ solution from a P^+^ type starting Si wafer, possess such a remarkable low reflectance and the electronic properties are affected by SiNWs’ homogeneity [23]. Otherwise, Hutagalung et al. also reported a low reflectance of less than 10% obtained by SiNW arrays synthesized via 1-MACE in an AgNO_3_ and HF solution at 60 °C from an n-type Si, whereas its I–V characteristics show linear ohmic behavior [24]. Therefore, careful production of SiNWs, by tuning the different parameters such as the etching time, etchant composition and concentration, etching temperature, and the starting Si wafer characteristics, is important to ensure good physical properties of SiNWs [23,24,25]. However, these previous works have neglected the PL study, as well as a deeper insight and understanding of the electrical properties via determining the electrical parameters is missing.

In this work, we contribute our recent results on the fabrication of optically and electrically-active SiNWs which exhibit a strong PL emission, remarkable antireflections properties, and interesting electrical properties. First, a detailed explanation of the fabrication process is reported and then vertical aligned SiNWs with relatively high crystallinity were obtained through the 2-MACE method. The effect of etching time key parameter on SiNWs’ filling ratio and length, optical, and electrical properties were investigated and evaluated. A broadband PL emission and low reflectance from these wires were obtained. Later on, the I–V characteristics and the electrical parameters were carefully determined and studied. Our findings could consider the optimized SiNWs as a promising candidate in PV and optoelectronic applications due to their unique structural, optical, and electrical properties.

## 2. Materials and Methods

### 2.1. Reagents and Materials

The chemical reagents used in this work for the cleaning or etching process such as acetone, ethanol, isopropanol, hydrofluoric acid (HF, 40%), silver nitrate (AgNO_3_), hydrogen peroxide (H_2_O_2_, 35%), and nitric acid (HNO_3_, 65%) were purchased from Sigma-Aldrich (Madrid, Spain). All of them were used without any purification. The single-side polished p-type silicon wafers were purchased from Siltronics (Archamps, France) and finally deionized water used in all the experiments was supplied by local sources.

### 2.2. Samples Preparation

SiNW arrays were synthesized by the Ag-assisted chemical etching method of (100) oriented p-type silicon wafers with the resistivity of 1–20 Ω cm. The fabrication process is as follows: (1) The silicon wafers were sequentially cleaned in acetone, ethanol, isopropanol, and deionized water (DI) in an ultrasonic bath for 15 min each. The cleaned wafers were then immersed in diluted HF for 3 min. (2) Silver nanoparticles were deposited onto the Si substrate using an aqueous solution composed of 4.8 M HF and 0.035 M AgNO_3_ for 1 min. (3) Silicon wafers covered with AgNPs were dipped into the etching solution of 4.8 M HF and 0.5 M H_2_O_2_ at room temperature during 20 (sample S1), 40 (S2), 60 (S3), 80 (S4), 100 (S5), and 120 min (S6). (4) The resulting samples were rinsed with DI and then immersed in nitric acid for 15 min to remove the silver nanoparticles and dendrites. Finally, the as-formed homogeneous black SiNWs were washed again with DI and dried with nitrogen.

### 2.3. Characterizations

The samples were studied using several techniques. The morphologies of the synthesized SiNWs were characterized using scanning electron microscopy (SEM, FEI Verios 460, FEI Europe B.V., Eindhoven, Netherlands). The observations were performed in a top view, cross-section, and 30° tilt view. X-ray diffraction (XRD) measurements were performed using an automated Bruker D8 advance X-ray diffractometer (Bruker, Karlsruhe, Germany) with Cu Kα (λ = 1.54 Å) in 2θ ranging from 20 to 80°. Photoluminescence spectroscopic analyses of SiNWs were made with a 405 nm laser wavelength and all the measurements were done at room temperature (RT). Moreover, we have used an analytical model to deduce the SiNCs size through PL spectra of the fabricated samples. The FTIR analyses were taken on an absorbance mode using Bruker IFS66v/s FTIR spectrometer (Bruker, Karlsruhe, Germany) and investigated in the 400–4000 cm^−1^ range with a step of 4 cm^−1^. Reflectance measurements were performed via Perkin Elmer Lambda 950 spectrophotometer (Perkin Elmer, Inc., Waltham, MA, USA). The current–voltage (I–V) measurements were measured via Keithley 2400 source meter (Keithley, Austin, TX, USA) in the dark and at room temperature.

## 3. Results and Discussion

### 3.1. Detailed Mechanism of SiNWs’ Formation

**Figure 1:**
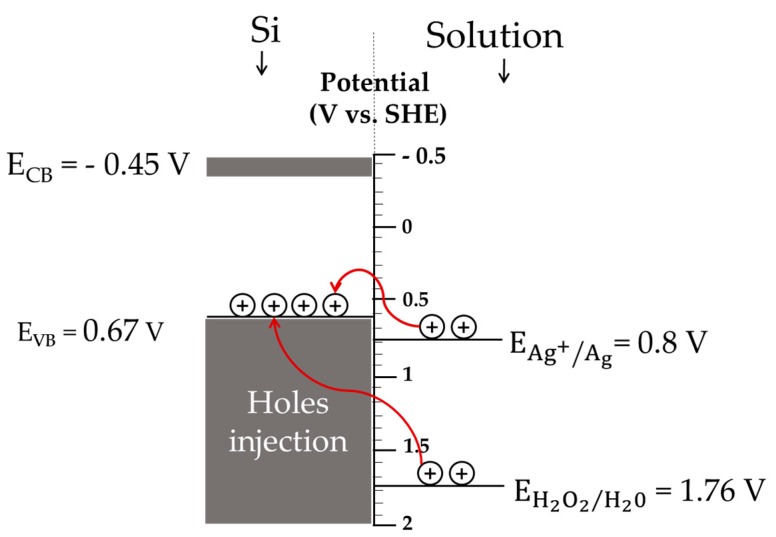
Schematic of the potential relationship between bands in a silicon (Si) substrate, Ag^+^/Ag, and H_2_O_2_/H_2_O redox pairs.

Two-step MACE was introduced to prepare vertically aligned SiNWs which is a simple, reproducible, and inexpensive process using Ag catalysts in the HF/H_2_O_2_ etching agent. To better understand SiNWs’ formation mechanism, the properties of Si, Ag^+^, Ag, and H_2_O_2_ in HF solution should be understood. Figure 1 shows a scheme of the potential distribution of Si, Ag^+^/Ag, and H_2_O_2_/H_2_O redox pairs in HF solution when the energy is referred to the standard hydrogen electrode potential (SHE).

The energies of the valence band (VB) and conduction band (CB) of Si are 0.67 and −0.45 eV, respectively while the potential energies of the redox pairs of Ag^+^/Ag and H_2_O_2_/H_2_O are 0.8 and 1.76 V, respectively [26].

**Figure 2:**
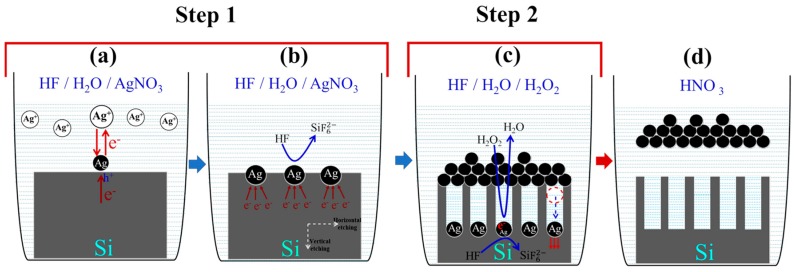
Schematic illustration of the formation mechanism of silicon nanowires (SiNWs) via two-step MACE. (**a**) Reduction of Ag^+^ ions and formation of Ag nuclei at the Si surface. (**b**) Further silver nuclei growth, oxidative dissolution of Silicon atoms, and production of porous layer. (**c**) Vertical propagation of silver nanoparticles and faster etching leading to SiNWs formation. (**d**) Silver removal and vertical aligned SiNW arrays production.

The growth mechanism of SiNW arrays was elucidated in this section and depicted in Figure 2.

After the cleaning stages, 2-MACE consists first of all in dipping the silicon wafers in a HF/AgNO_3_ aqueous solution producing AgNPs deposition. Indeed, the AgNO_3_ metallic salt come apart in the HF aqueous solution to yield metal ions Ag^+^. The Ag^+^ ions close to the Si surface extract electrons from the VB of Si resulting in a small silver nuclei on the surface of the silicon (Figure 2a). These electrons transfer continues to take place as Ag is more electronegative than Si leading to a large silver nuclei growth and then a formation of Ag-nanoclusters or a continuous silver layer. Furthermore, the excess of electrons extract forms an accumulation of holes beneath and around the catalyst.

This leads to silicon oxidation followed by HF dissolution into silicon hexafluoride ions ($\mathrm{Si}F_{6}^{2-}$) while unveiling a newly exposed Si coming in contact with the Ag catalysts which will be further etched in the second step of the continuous MACE process. The synchronized mechanism of Ag^+^ reduction (cathodic process) and silicon oxidation (anodic process) can be described by the following equations:

cathode reaction Ag^+^ + e^−^ → Ag E^0^ = 0.79 V (vs. SHE) (1)

(2) $$\mathrm{anode}\;\mathrm{reaction}\;\mathrm{Si}+6\mathrm{HF}\to \mathrm{Si}F_{6}^{2-}+4e^{-}+6H^{+}$$

(3) $$\mathrm{overall}\;\mathrm{reaction}\;\mathrm{Si}+6\mathrm{HF}+4{\mathrm{Ag}}^{+}\to \mathrm{Si}F_{6}^{2-}+4\mathrm{Ag}+6H^{+}$$

At a certain while, the hole injection rate attenuates because almost all Ag^+^ ions in the vicinity of the silicon surface are reduced into Ag. Therefore, silicon oxidation slows down causing a decrease in the Si etching rate which requires a complementary hole injection species to permit the continuation of the silicon nanostructuring. Subsequently, H_2_O_2_ is added to the etching solution because it is a strong oxidant allowing a hole injection instead of Ag ions. When the silicon wafer covered with the silver, dendritic layer was put into the HF/H_2_O_2_ aqueous solution, the second step in MACE mechanism which corresponds to the etching process starts to take place. Since the redox potential of H_2_O_2_ is more positive than that of Ag, H_2_O_2_ captures electrons from the previously nucleated Ag particles and reduces to H_2_O. Hence, Ag is oxidized immediately to Ag^+^, this oxidation allows the solution feeding with Ag^+^ ions which enhances the hole injection. The silicon is then locally oxidized into SiO_2_ and dissolved simultaneously by HF, so the AgNPs sink downwards vertically along the (100) direction in the pits thus formed leading to the generation of vertical SiNWs (Figure 2c). This is contrary to porous silicon (PS) which displays a porous surface morphology due to the absence of AgNO_3_ in the etching solution and the slow etching rate. The redox reactions taking place during etching process can be described as follows [27,28]:

cathode reaction H_2_O_2_ + 2e^−^ + 2H^+^ → 2H_2_O E^0^ = 1.76 V (vs. SHE) (4)

anode reaction Si + 2H_2_O → SiO_2_ + 4H^+^ + 4e^−^ (5)

(6) $${\mathrm{SiO}}_{2}+6\mathrm{HF}\to \mathrm{Si}F_{6}^{2-}+2H_{2}O+2H^{+}$$

(7) $$\mathrm{Overall}\;\mathrm{reaction}\;\mathrm{Si}+2H_{2}O_{2}+6F^{-}+4H^{+}\to \mathrm{Si}F_{6}^{2-}+4H_{2}O$$

Using Reaction (7), the potential ΔE of etching process is as follows:

(8) $$\Delta E=\Delta E^{0}-\frac{0.059}{4}\left\{\log\frac{\left[5{\mathrm{iF}}_{6}^{2-}\right]}{{\left[H_{2}O_{2}\right]}^{2}{\left[H^{+}\right]}^{4}{\left[F^{-}\right]}^{6}}\right\}$$

According to this equation, the increase of H_2_O_2_ and HF concentrations enhance the reaction potential with an increase in the etching rate. However, the etching rate may not increase infinitely and it is preferable to choose an appropriate range of etchant concentration to ensure good quality SiNWs formation. It is suggested that the self-grown SiNCs on SiNWs form due to sidewall etching such us the extra Ag^+^ ions spread through the nanowires and trap electrons from the sidewalls leading to a lateral etching of SiNWs and consequently formation of porous SiNWs decorated with SiNCs [20]. Ultimately, the removal of AgNPs and dendrites by immersing the samples in an HNO_3_ aqueous solution reveals highly oriented one-dimensional (1D) SiNWs. The corresponding reaction is expressed as:

3Ag + 4HNO_3_ → 3AgNO_3_ + NO + 2H_2_O (9)

### 3.2. Etching Time Effect on Morphology of SiNW Arrays

**Figure 3:**
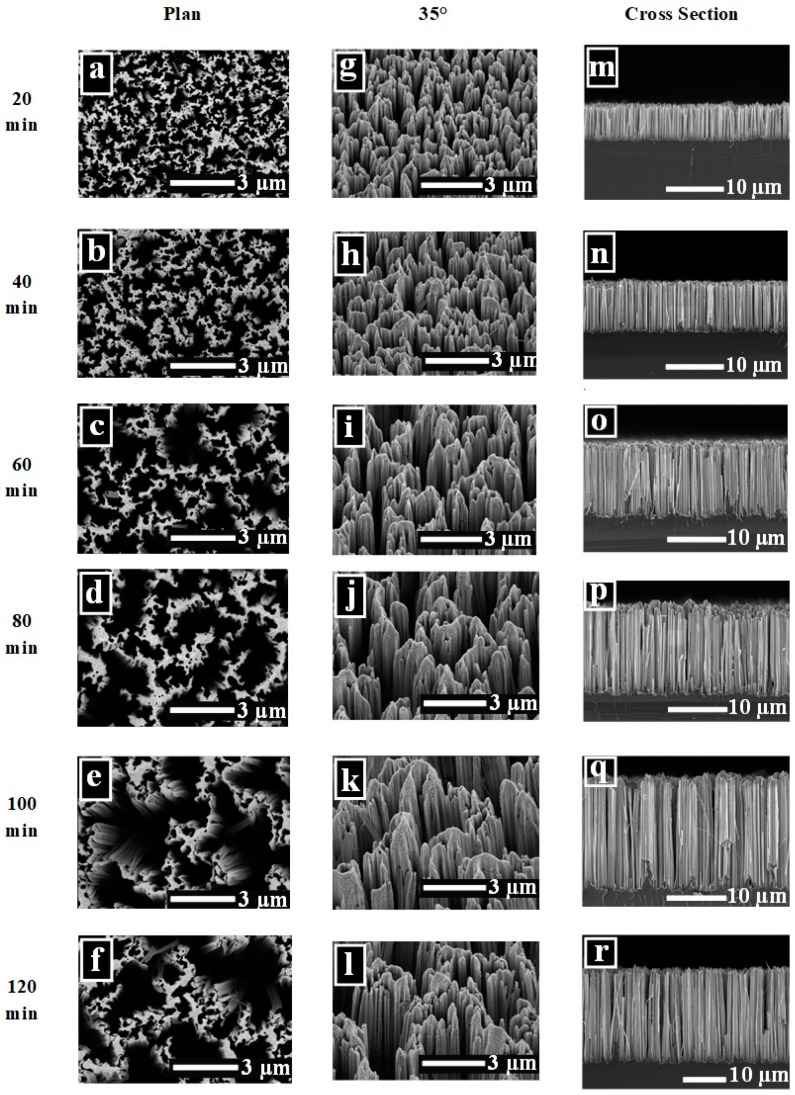
SEM images of SiNWs etched at different durations showing top view surface (**a**–**f**), tilt view 35° (**g**–**l**), and cross-sectional (**m**–**r**).

**Figure 4:**
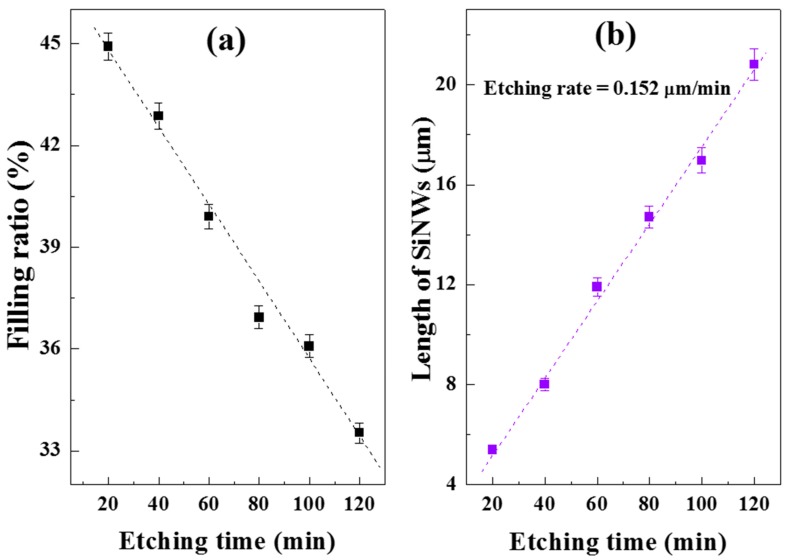
Variation of the (**a**) filling ratio and (**b**) length of SiNWs as a function of etching time.

In order to investigate the effect of etching time on SiNWs morphology and structure, the AgNO_3_, HF, and H_2_O_2_ concentrations were fixed in both deposition and etching steps. SEM images (top view, cross-section, and tilt view at 30°) of the as-prepared SiNWs etched in different times (20–120 min), are shown in Figure 3. The SiNWs dependence on etching time was explored. Forest-like SiNW arrays can be noticed from the top view images (Figure 3a–f). The NWs etched during short etching time are isolated from each other. However, the tips of the nanowires congregate together with time increment to form bundles. This bundle-like structures distribute uniformly on the whole wafers and could be confirmed from the tilt view images. The possible reasons behind this conglomeration may be attributed to Van der Waals attraction between the nanowires [29,30] as well as to the increase in the length of SiNWs that likely causes them to bend to form bouquets under the action of gravity. Otherwise, it was also reported that some nanowires could remain unattached due to inhomogeneous etching induced by a random silver particle distribution [31]. The calculated values of average volume filling ratio (VFR) at the air/SiNW arrays interface versus etching time is plotted in Figure 4a and it showed the decreasing of VFR when the etching time increase. For example, VFR is 0.45 in dense SiNWs at 20 min decreases to 0.33 in convergent SiNW arrays at 120 min of etching time. This is due to the bundle-like structures of the wires in prolonged etching times. Chang et al. [32] reported a similar variation in VFR, however, as a function of AgNO_3_ concentrations.

Vertically aligned SiNWs with good uniformity were clearly seen in the cross-section images (Figure 3m–r). The length of nanowires significantly increases from 5.48 to 20.84 μm with increasing the etching from 20 to 120 min whereas the wire diameter is approximately in the range of one hundred to a very few hundreds of nanometers.

Hence, the linear relationship of SiNWs length versus etching time plotted in Figure 4b gives an etching rate close to 0.152 µm/min.

This linear relation has been investigated in literature but with different etching rate values [2,18,33]. The possible reasons behind this increase of SiNWs length with etching time was ascribed to the fact that etching was given more time to proceed and the solution had enough oxidizing species to oxidize and dissolve the formed SiO_2_. We retain that doubling the etching time would not necessarily double the length of SiNW [34,35]. From tilt view images, we can observe that SiNWs are covered by numerous porous structures especially on their tips due to the additional etching pathways via the re-nucleation of the AgNPs throughout the SiNWs.

These porous structures are the origin of quantum confinement effects owing to their small dimensions. SiNW arrays grown by MACE usually show these porous structures called silicon nanocrystals.

**Figure 5:**
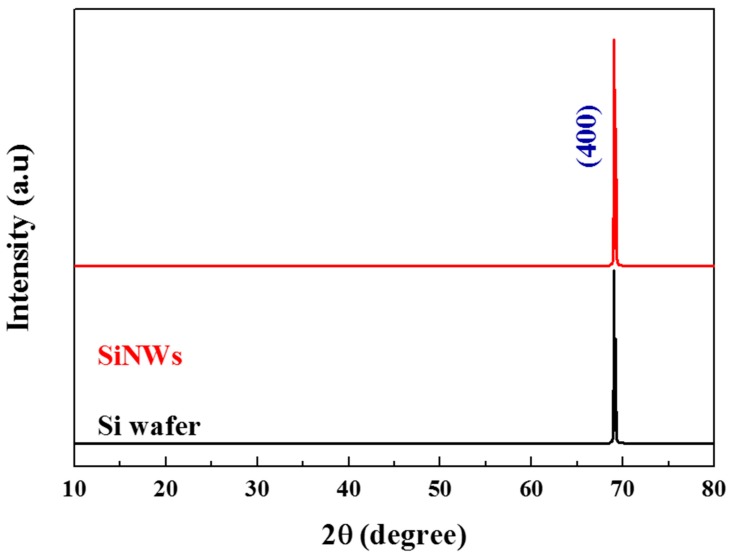
XRD patterns of silicon nanowires and untreated Si wafer.

Figure 5 displays XRD patterns of untreated Si wafer and SiNWs. A unique sharp peak at 69° is observed for both samples, which is indexed to a (400) silicon plane. However, the silicon nanowires give a high peak intensity than that of Si wafer. This suggests high quality crystalline nanowires.

On the other hand, no characteristic peaks of silver are observed in the XRD pattern which confirms the completely removal of silver nanoparticles by nitric acid.

### 3.3. Photoluminescence Spectroscopy

**Figure 6:**
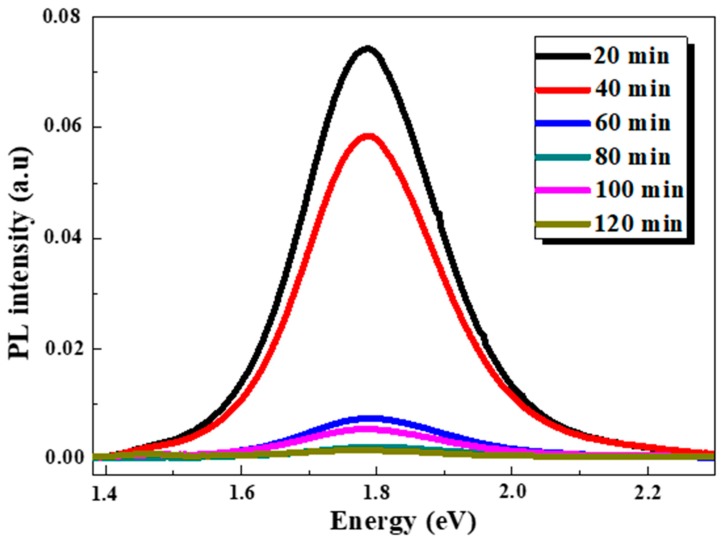
PL spectra of SiNWs synthesized at different etching times.

The room temperature PL measurements of the as-synthesized samples were carried out in order to study the etching time dependency on photoluminescence properties. The SiNWs PL spectra ranging from 1.4 to 2.3 eV are shown in Figure 6.

Broad PL emission bands were recorded with a maximum around 1.78 eV and a full width at half maximum (FWHM) of 0.24 eV. Their shape is a gaussian and did not present any significant shift. However, one can see a clear degradation in the PL intensity when increasing etching time.

**Figure 7:**
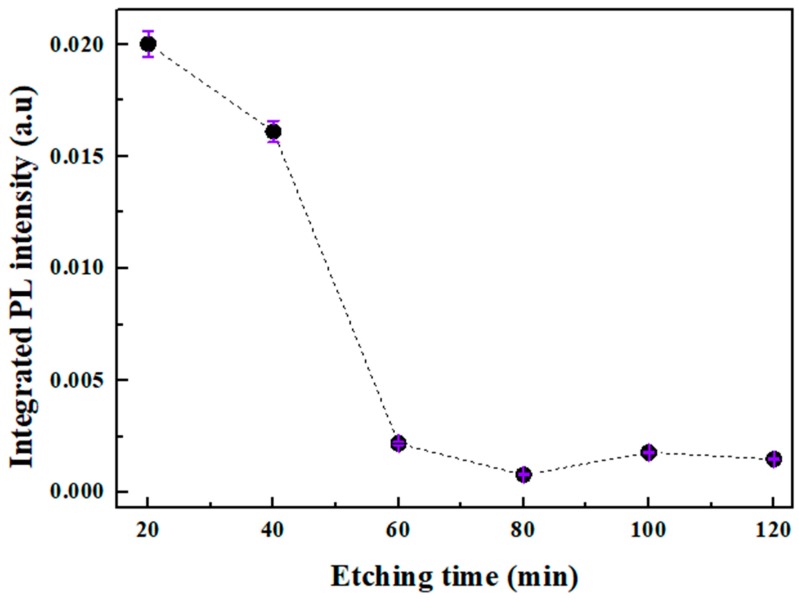
Variation of integrated PL intensities versus etching duration.

The highest PL intensity was obtained for 20 and 40 min which are almost 26 times stronger compared to the prolonged etching time. Therefrom, the opposite trend between the integrated PL intensity and etching duration is plotted in Figure 7.

To explain the origin of this PL emission, several mechanisms have been proposed including the quantum confinement (QC) effects and the presence of defects in an SiO_x_/Si interface and/or in the surface of the oxide related to the Si–O–Si bonds [36,37]. However, the QC effects, which enhance the radiative recombination of excitons, remain the most approved model which dictates that the crystallite Si size should be less than the Bohr radius of the free exciton of bulk silicon [17]. In the present work, the SiNW diameters are within one hundred to a very few hundred of nanometers, these values are much larger than the Bohr radius of excitons (5 nm) present in Si crystals. Taking into account the indirect band gap of SiNWs and their large diameters, the observed visible PL is unlikely to originate from the SiNWs and the origin of this light emission must be more complex. This is dissimilar to one earlier report that attribute the visible PL to the radiative recombination at the SiNWs itself [38]. On the other hand, it is widely reported that the SiNWs are decorated with self-grown silicon nanocrystals whose dimensions are smaller than the exciton Bohr diameter in silicon. These SiNCs cause a robust quantum confinement leading to the appearance of observed PL at room temperature [2,3,4,20]. In the current study, the nanocrystal size resulted from the spectral position of the PL peak by using the equation described below:

(10) $$E=E_{g}+\frac{c}{d^{\alpha }}$$

where E is PL peak position (eV), E_g_ is the band gap of c-Si (1.12 eV), *d* is the SiNC size (nm), α and C are constants. Several works have reported different values of these constants from the SiNWs/NCs [19,39,40,41]. Comparison of SiNCs size values obtained by different parameters from earlier reports of the literature is investigated and the evaluated NC-sizes are summarized in Table 1. One can see clearly that all the estimated NCs diameter values are less than 5 nm and close to 2~4 nm regardless of the formula used (Table 1). According to these below values, we can assert that in these samples, the occurrence of QC in SiNCs is the origin of the recorded PL emission. Note that similar SiNCs size and PL peak position have been recently carried out [2,3,41] due to the known dependence of a SiNC band gap on its size.

Interestingly, the compatibility between the samples (S1–S6) in terms of peak position, FWHM, NCs size, and the variation of the PL intensity indicate that these latter derive from different size distribution and densities of the inseparable set of SiNWs-NCs. This is confirmed by the reduction of SiNWs amount as seen above in the SEM images and more precisely by the reduction of the volume filling ratio over etching time. These latter indicate that the monotonous decrease of the PL intensity over etching duration is interpreted as restriction and diminution of excitation volume which in turn is proportional to the amount of effective light-emitting crystallites [17,41,42].

Therefore, an appropriate geometry is required to ensure PL and our findings demonstrate that shorter SiNWs are much more optically active. It is worth noting that the light emitted from the SiNW arrays upon laser irradiation is visible to the naked eye and appears orange obviously for the samples corresponding to 20 and 40 min etching time. This emission is attributed to the radiative recombination of excitons in small SiNCs present in the nanowire sidewalls in terms of a QC model as explained above.

### 3.4. FTIR Analysis

**Figure 8:**
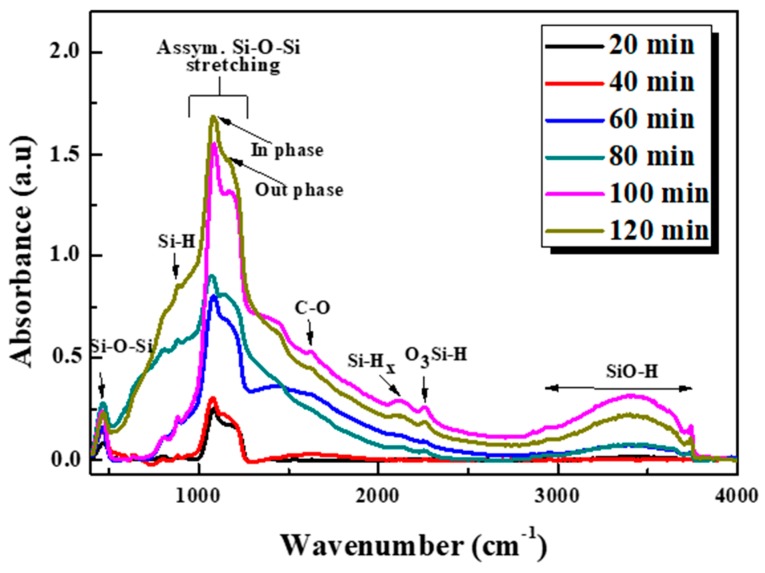
FTIR spectra of SiNWs obtained at different etching time.

FTIR spectroscopy was used to study the surface composition of the as prepared SiNWs at different etching durations. An untreated silicon substrate was utilized as background to the measurements and the spectra were taken in absorption mode in the 400–4000 cm^−1^ spectral range as shown in Figure 8.

All the spectra are closely similar with an increase in the absorbance bands intensity versus increasing etching time or otherwise with increasing SiNWs length. The most intense broad peak appears in the region between 1000–1300 cm^−1^ and is assigned to the Si–O–Si asymmetric stretching (AS) vibrations. This observed signal can be classified into a strong band at 1080 cm^−1^ attributed to AS_1_ vibration mode and a shoulder at 1200 cm^−1^ related to AS_2_ vibration mode. AS_1_ results from the motion of the adjacent oxygen atoms moving in phase with one another while at AS_2_ the oxygen atoms move 180° out of phase with one another. The appearance of this shoulder is a feature of the IR spectra of SiO_x_ material where x value equals to one or higher [43].

**Figure 9:**
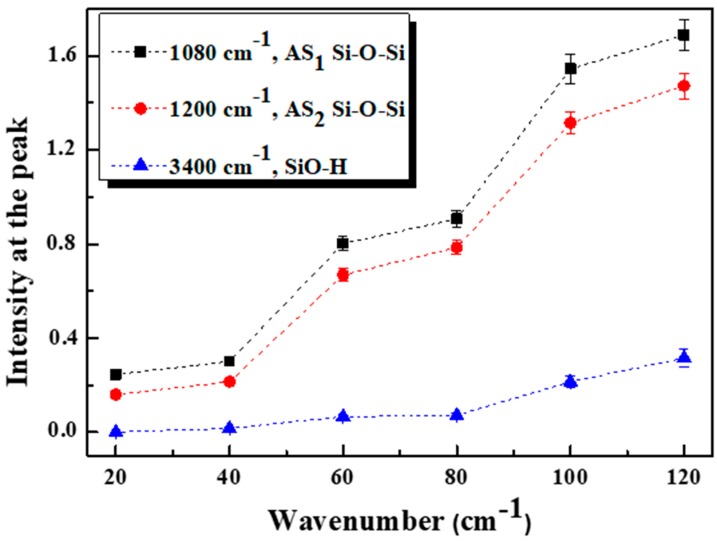
Evolution of peaks intensity of dominant FTIR bands versus etching time.

These Si–O–Si bonding, introduced during the electrochemical etching, were found to be more popular with increasing etching time. Hence, Figure 9 presents the evolution of peaks intensity of dominant bands existing in the spectra.

This increase of intensities indicates the continuity of chemical etching even during extended durations and then the formation of Si–O radicals becomes consistent when rising the immersion duration in HF/H_2_O_2_. Here, it involves that the charge transfer faces some difficulty in order to establish an electrical equilibrium leading to a decay in etching rate [44]. This behavior is in accordance with PL results and hence we could say that PL deterioration in higher etching time is related to the change in the distribution and density of the inseparable set of SiNWs-NCs owing to this etching rate decay leading to a restriction of excitation volume and therefore reduction of luminescent crystallites’ density.

The broad peak at 465cm^−1^ was related to Si–O–Si, while the broad band within 3000–3700 cm^−1^ is attributed to the stretching of the O−H bonds in SiOH groups and surface adsorbed H_2_O. Whereas the narrow band at 3747 cm^−1^, is attributed to the stretching mode of surface free OH groups. The weak IR absorption peaks situated at around 900, 2125, and 2258 cm^−1^ corresponds to Si–H bending vibrations, Si–H_x_ bonds and to Si–H stretching mode in O_3_–SiH, respectively. Finally, the tiny absorption band barely detectable at 1630 cm^−1^ is attributed to C−O bonds which mainly came from the SiNWs surface contamination from environment and can hardly be avoided [2,21,28,43]. We notice that the surface states assigned to Si−O and Si−H chemical bonds are popular in all samples.

### 3.5. Reflectance Analysis

**Figure 10:**
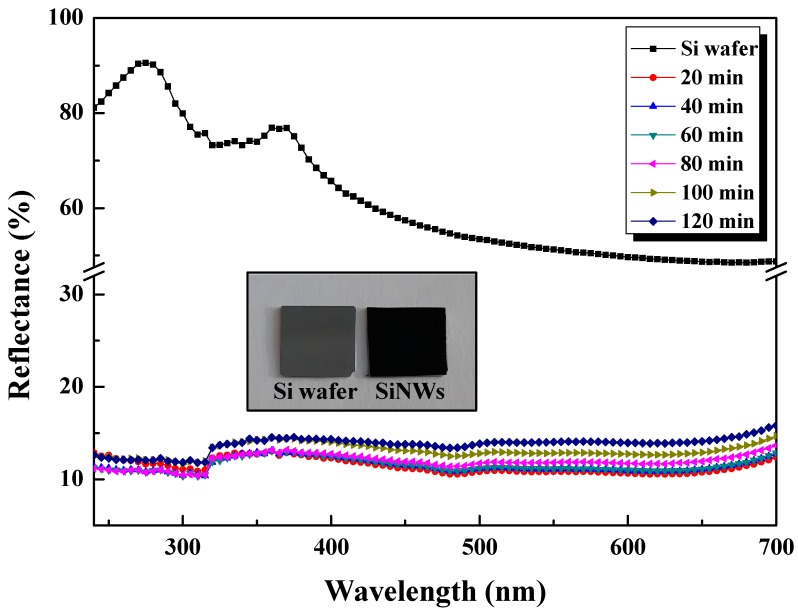
Reflectance spectra of SiNWs with various lengths etched during different times and corresponding Si wafer. The inset displays images of Si wafer and SiNWs.

Figure 10 shows the reflectance spectra of the untreated Si wafer and the synthesized SiNWs with various heights over the wavelength range of 250–700 nm which covers the main spectral irradiance of sunlight that is useful for Si solar cells.

When compared to the untreated bulk Si, all the etched samples show a significant lower reflectivity which is consistent with the colour changes from metallic grey to dark black as shown in the inset. The average reflectance for bulk Si is about 50%~90% in the UV–visible range while it reaches less than 15% for all the etched samples. Hence, such nanowires reduce reflection losses and further enhance the carrier collection which is extremely beneficial for developing high efficiency solar cells requiring good antireflective properties.

The sharp suppression in the reflectance is ascribed to three important factors: (i) The gradual variation towards the refractive index from air (n ≈ 1) to SiNWs and to Si substrate (n ≈ 3.42), (ii) the tapered morphology of silicon wires leading to light trapping due to the multiple reflections back and forth in the inner surface and (iii) the sub-wavelength structures for additional light trapping within the NWs [17,33,45]. The observed peaks of Si wafer at 275 and 367 nm come from the inter-band transitions of Si [32,45]. In addition, as readily seen in the figure, all the reflectance values of SiNW arrays with various lengths are almost close and low (9–15%) owing to the tapered NWs structure. The slightly higher reflectance observed for prolonged etching time is attributed to the coalescence of NWs at the apex and bundles formation as shown above in SEM images [33,45].

### 3.6. I–V Measurements

**Figure 11:**
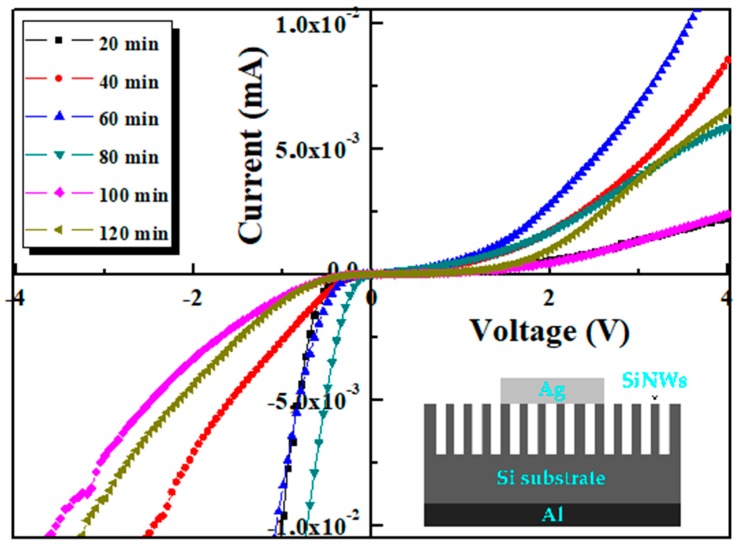
Curves of the as-prepared heterojunction based on SiNWs synthesised at various etching times. The inset displays a schematic illustration of Ag/SiNWs/Si/Al device.

Electrical properties of SiNWs samples with various lengths of nanowires are studied through measurement of current–voltage (I–V) characteristics at room temperature and in the dark. First, Schottky contact was formed between metal and semiconductors (Al/Si and Ag/SiNWs). The I–V measurements of Ag/SiNWs/Si/Al structure from −4 to 4 V are depicted in Figure 11.

All the curves corresponding to NWs show a diode-like nature with an obvious rectifying behaviour. Interestingly, the junctions based on these SiNWs etched at different etching time display considerably the same properties at different areas on the junction. This latter observation highlights the uniformity of the junctions and could be supported by the distribution of a uniform large area of SiNWs on the whole wafers as shown above in the SEM images. On the other hand, the fabrication of other devices with maintaining the same conditions pointed out an electrical reproducibility property of the junctions. One can note that SiNWs fabrication with good uniformity/reproducibility are a MACE feature [4,34]. It is worth noting that the observed rectifying behaviour of these SiNWs is similarly shown in the literature for porous silicon [42,46,47,48]. Importantly, the nonlinear I–V characteristics and the rectifying event are mainly controlled by the SiNW layers and ascribed to Ag/SiNWs:p-Si since the Si/Al interface was proved to be ohmic [47,48]. The electron flow into the SiNW layers from Ag and recombine with holes, leaving negatively charged electrons in SiNWs which leads to the N-type role of SiNWs with respect to Si. Thus, the formed depletion layer causes the rectifying behaviour. Another reason is that the quantum confinement coming from the SiNCs in the SiNWs increases the band gap in the SiNWs side which creates a potential barrier leading to the diodic behaviour [49,50]. It is clear to notice from the I–V curves the increase of rectification extent (current intensity) in the forward bias with etching time or in other words with SiNWs’ length until reaching a threshold time (length) which corresponds to 60 min (≈12 µm), then the current intensity decreases. Therefore, we can assume that the electrical transport properties of the device are governed by SiNWs’ contribution. The current decrease for samples with higher etching times is attributed to the increase of SiNWs length. The wires, acting as carries trapped, will increase and form a high resistive region which lead to the decrease of the current flowing through the SiNW layers.

Therefore, a well-defined moderate length of SiNWs is required to ensure better electrical properties. Electrical parameters of the device are obtained using the conventional thermionic emission model (TE) as expressed:

(11) $$I=I_{s}\left(\exp\left(\frac{q\left(v-{\mathrm{IR}}_{s}\right)}{\eta \mathrm{kT}}\right)-1\right)$$

where q is the electronic charge, η is the ideality factor, R_s_ is the series resistance, k is the Boltzmann constant, T is the absolute temperature, and I_s_ is the reverse saturation current given by:

(12) $$I_{s}={\mathrm{aA}}^{*}T^{2}\exp\left(\frac{-{q\varphi }_{b}}{\mathrm{kT}}\right)$$

Herein, a is the diode area, A* is Richardson constant (≈32 A cm^−2^ K^−1^ for p-type Si) and $\varphi_{b}$ is barrier height. As eV >> ηKT at room temperature, Equation (11) can be rewritten as:

(13) $$I=I_{s}\exp\left(\frac{q\;V}{\eta \mathrm{kT}}\right)$$

**Figure 12:**
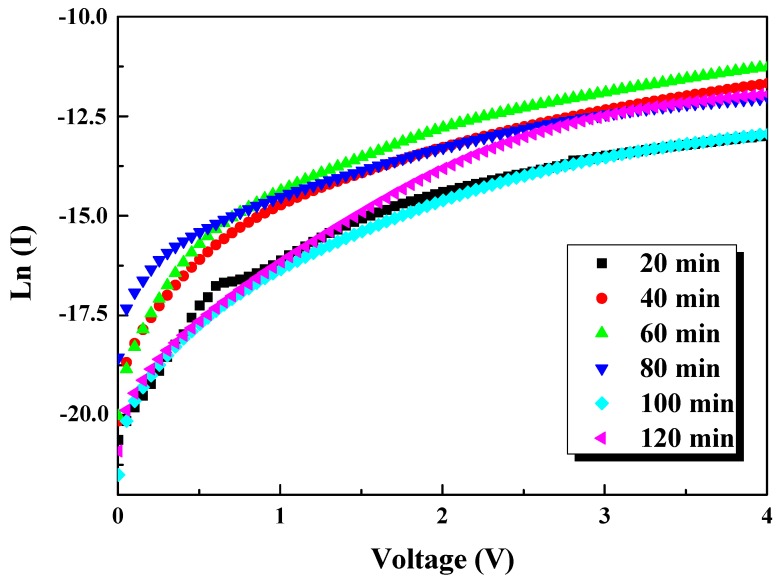
Logarithm of forward current versus voltage (Ln (I) vs. V) curves of SiNWs synthesized at different etching times.

The ideality factor η was estimated from the slope of the linear region of the plot of ln(I) vs. V (Figure 12) based on Equation (13). The saturation current was derived by extrapolating ln(I) vs. V plot to V = 0 while $\varphi_{b}$ was calculated through Equation (12).

In order to have more accurate ideality factor values owing to the non-linear part of ln(I) vs. V plots and to determine the series resistance (R_s_), the Cheung’s functions were utilized [51,52]. Thus,

(14) $$\frac{\mathrm{dV}}{d\left(\mathrm{LnI}\right)}=R_{s}I+\frac{\eta \mathrm{kT}}{q}$$

**Figure 13:**
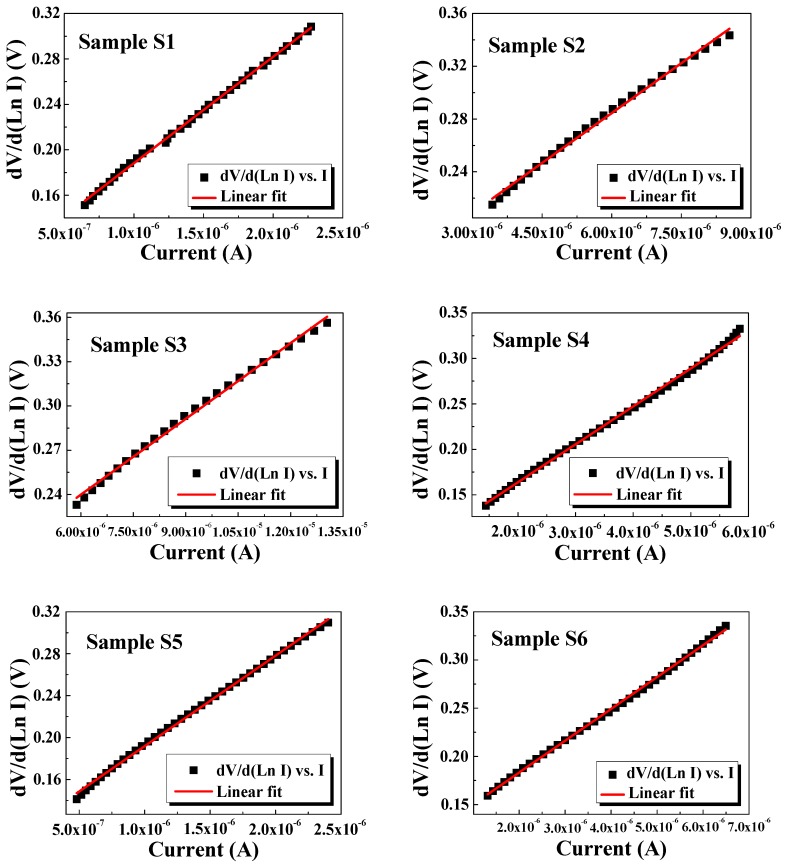
Plots of dV/d(LnI) vs. I of SiNWs samples (S1–S6).

Figure 13 presents the experimental $\frac{\mathrm{dV}}{d\left(\mathrm{LnI}\right)}$ vs. I plots, the R_s_ and η were determined as the slope and y axis intercept, respectively.

The deduced values of Is, η, $\varphi_{b,}$ and $R_{s}$ for samples S1–S6 are summarized in Table 2 and show a notable dependence of SiNWs’ length. According to the formula in Equation (11), high I require high Is and small η and R_s_. The ideality factor values estimated from Cheung’s functions are nearly matched with I–V method values. These relatively high values could be attributed to series resistance, barrier inhomogeneities, interfacial defects, or existence of an oxide layer spontaneously produced during the synthesis [51,52]. These latter give sense to the deviation from ideality and η deterioration especially for higher etching times. A similar trend is noticed for barrier height. The results point out that the electrical parameters depend upon the thicknesses of SiNWs and the use of shorter SiNWs not exceeding 12 µm (corresponding to 40 min etching time) can result in better electrical properties. One can note that these η values are lower compared to those reported on porous silicon [47]. This is suggested to be related to the unique properties of SiNWs compared to PS which is promising for optoelectronic devices application.

## 4. Conclusions

In summary, optically and electrically-active SiNWs with strong PL emission, remarkable antireflections properties, and interesting electrical properties are successfully synthesized via 2-MACE method. First, an expanded explanation of the fabrication process is reported and then a systematic study is presented to visualize the effect of varying etching times on morphological, structural, optical, and electrical properties of SiNWs. The NWs length was increased by increasing etching time with a rate about 152 nm/min, whereas the wires filling ratio decreased. An analytical model is utilized to calculate the sizes of the self-grown silicon nanocrystallites which are the origin of the observed photoluminescence emission. FTIR spectroscopy supports PL results and confirms that the PL intensity degradation for expanded durations is owing to the restriction of excitation volume and therefore reduction of luminescent SiNCs. Therefore, appropriate SiNWs in terms of length and geometry is strongly required to ensure good PL. When compared to the silicon wafer, the formed SiNWs demonstrate a strong decrease of the reflectance to 9–15%. This strong reduction of reflectance certifies that SiNWs are an excellent candidate for photovoltaic cells. I–V characteristics revealed that the rectifying behaviour of the uniform-reproducible junctions and the diode parameters (Is, n, φ_b_, and R_s_) calculated from conventional thermionic emission and Cheung’s model are found to depend significantly on the geometry of SiNWs. In brief, we deduce that etching time or otherwise SiNWs’ length plays a key role on optical and electrical properties of SiNWs. Hence, judicious optimisation of these latter parameters is robustly required for better SiNWs’ physical properties. Our findings demonstrate that shorter SiNWs are much more optically and electrically effective which pave the way for its application in the field of optoelectronic devices and solar cells.

## Figures and Tables

**Table 1:** Comparison of the SiNCs sizes value estimated from Equation (10) for different reports of the literature.

Constants	Valenta et al. [39]	Yan et al. [40]	Gonchar et al. [41]	Bahera et al. [19]
C (eV nm^−2^)	2.49	4	3.73	2.4
α	0.91	1.4	1.39	1.7
d (nm)	4.25	3.59	3.45	2.12

**Table 2:** Various electrical parameters determined by conventional TE and Cheung’s model.

	Ln (I) vs. V (TE model)			Cheung’s Functions	
**Etching Time**	**I_s_ (µA)**	**η**	$\varphi_{b}$ **(eV)**	**R_S_ (k** **Ω** **)**	**η**
20 min	0.211	5.91	0.806	93.65	3.65
40 min	0.495	5.31	0.790	32.10	3.56
60 min	0.695	4.38	0.771	25.06	2.60
80 min	0.566	6.17	0.768	41.75	3.10
100 min	0.239	6.05	0.801	86.33	4.08
120 min	0.224	6.41	0.804	33.01	4.53
